# Infection Control Improvement of a Negative-Pressurized Pediatric Intensive Care Unit

**DOI:** 10.3390/healthcare9111500

**Published:** 2021-11-04

**Authors:** Fujen Wang, Indra Permana, Citra Chaerasari, Bivas Panigrahi, Dibakar Rakshit

**Affiliations:** 1 Department of Refrigeration, Air Conditioning and Energy Engineering, National Chin-Yi University of Technology, Taichung 411, Taiwan; citra.chaerasari19@gmail.com (C.C.); bivas@ncut.edu.tw (B.P.); 2 Graduate Institute of Precision Manufacturing, National Chin-Yi University of Technology, Taichung 411, Taiwan; indra.refrigeration@gmail.com; 3 Department of Energy Science and Engineering, Indian Institute of Technology Delhi, New Delhi 110016, India; dibakar@iitd.ac.in

**Keywords:** infection control, isolation room, computational fluid dynamics, ventilation performance

## Abstract

The COVID-19 pandemic caused by the novel SARS-CoV-2 virus raises alarming concern around the healthcare facilities due to the significant increase in patient inflow. Negative-pressurized isolation rooms have been utilized in various health care facilities to isolate the patients from active community contact. Several studies have highlighted isolation rooms improvement. However, limited knowledge is available regarding the isolation room facilities for pediatric intensive care units (PICU) to accommodate more than one pediatric patient. In this aspect, this study investigates a negative-pressurized isolation facility in PICU with minimal design modifications with the possibility that it can accommodate more than one pediatric patient. The field measurement tests were conducted to ensure the design compliance of Taiwan CDC. Then, computational fluid dynamics (CFD) was further utilized to numerically evaluate the HVAC system role and the ventilation performance towards infection control. A protected air-jet curtain system with a new ventilation layout was proposed through this study to enhance the protection for both pediatric patients and medical staff. The concentration decay was monitored and recorded within 900 s to evaluate the performance. The concentration can be reduced to 504 ppm for case 1, 620 ppm for case 2, 501 ppm for case 3, and 486 ppm for case 4. In addition, the injected bioaerosol particles could be well diluted dealing with two patients presents a good performance. The results revealed that this proposed configuration could feasibly accommodate two patients with a significant contamination control to protect the medical staff and patients.

## 1. Introduction

The outbreak of a new infectious disease called COVID-19 was declared a pandemic by World Health Organization (WHO). COVID-19 disease is caused by a novel coronavirus, SARS-CoV-2, which is plausibly transmitted through exhaled air from the respiratory tract of an infected person [1]. It could potentially carry pathogens that are responsible for the transmission [2]. US Centers for Disease Control and Prevention (CDC) developed and recommended isolation guidelines to prevent person–person transmission. In pediatric wards specifically for infectious disease, the demand for isolation beds may be ten-fold greater than for hospitalized adults [3]. Most pediatric wards have a limited number of single-occupancy rooms, which marked a problem when the demand for pediatric intensive care units (PICU) specifically for negative-pressurized isolation rooms gradually increased during the pandemic [4]. Children have special medical needs to provide critical care and infection control; therefore, a PICU isolation room is indispensable to a hospital unit. The general recommendation is to provide at least an isolation room for every ten beds or one for each pediatric unit [5]. CDC guidelines [6] recommend using a single negative pressure room for young children (0–14 years old) specifically for isolation precautions (airborne, droplet, or contact), except for children infected with the same pathogen that could get another alternative offer such as cohort nursing.

The PICU unit’s environmental system and facilities must conform to the standard and regulation depending upon the location. The fundamental guidelines for PICU, American Academy of Pediatrics (AAP) [7] stated that the room temperature must be adjustable between 22 and 25 °C with a relative humidity range of 30–60%. A minimum of 6 air changes per hour (ACH) must be supplied to the room. The ANSI/ASHRAE Standard-170 [8] also provides a general guideline on the ventilation system for the isolation room. Pressurization, ventilation, and HEPA filtration are the specific requirements that need to conform for the isolation room. Negative pressurization can be achieved through ventilation system control by exhausting more air, which is around 10% larger than the supply air volume [9]. Numerous international and professional organizations publish guidelines and recommendations for isolation rooms; therefore, the standard guidelines are different in every country. In this case, the isolation room simulation model is located in Taiwan. It must comply with the ventilation rate requirements between 8 and 12 ACH, and the differential pressure with its adjacent space should be at least 8 Pa [10]. The anteroom is recommended in each isolation room to minimize the spread of airborne infection [11]. HVAC system plays a significant role in increasing or reducing the spreading of infection in the indoor environment. The dispersion of droplets and aerosols has considerable relevance in the spread of this virus, often present in small particles with a diameter of less than 5 μm [12]. World Health Organization [13] has stated that particles with diameters less than 5 μm are called droplet nuclei (aerosol).

Computational fluid dynamics (CFD) is employed to simulate the airflow distribution pattern and model the motion of exhaled aerosol particles in the isolation room. These factors will determine the contaminants distribution and the airflow path of aerosol from the patients. Thus, it helps investigating the impact of isolation rooms occupied by two patients on ventilation and contamination control performance. Several experimental studies have been conducted through CFD numerical analysis related to a negative-pressurized isolation room. An experiment and simulation study was conducted by Liu et al. [14] on the innovative pediatric isolation bed. The concept was similar to protected occupied zone ventilation (POV) with air purification devices. The results showed that the child’s position should be placed near the air inlet hood with a velocity of 0.86 m/s to reduce cross-infection by air transmission in the pediatric ward. A recent study by Boro et al. [15] on the role of HVAC in the waiting room and patient room of children’s hospital focused on the diffusion of SARS-CoV-2. Through CFD simulation, it was revealed that higher airflow would reduce the contaminant concentration. It also leads to a substantial increase of turbulent air motions and spreading the droplets and aerosol throughout the room. An innovative local exhaust ventilation system could significantly reduce the droplets and contaminated air in the isolation room [16].

CFD simulation study experimentally based on different location exhaust air grille was conducted by Cho [17]. The placement of two exhaust air grilles beside the patient’s head was the best location to provide a direct exit path for the airborne contaminant. Jacob et al. [18] and Khankari [19] also demonstrated that the location of supply air across the patient and exhaust grille over the patient head could work collaboratively to establish effective contaminant control. Another experimental and simulation study by Cao et al. [20], based on protected occupied zone ventilation (POV), investigated the improvement of the ventilation quality and prevention of cross-contamination by using a downward plane air-jet. Even though many studies have been conducted in the isolation room, there is still limited knowledge of isolation room dealing within pediatric intensive care units occupied by more than one patient. In this study, numerical simulation and field measurement test was conducted in a newly constructed PICU isolation room located within a hospital in Taiwan. The simulation results were investigated extensively on the airflow pattern and contamination concentration to improve the ventilation performance and contaminant control.

## 2. Negative-Pressurized Pediatric Intensive Care Unit

### 2.1. System Description

**Figure 1:**
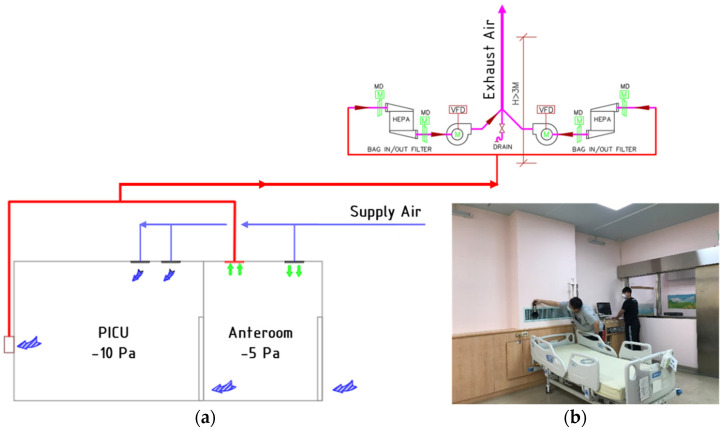
The investigated pediatric intensive care unit isolation room: (**a**) schematic diagram of the HVAC system; (**b**) snapshot.

The schematic diagram of the negative-pressurized pediatric intensive care unit (PICU) is shown in Figure 1. Isolation rooms in health care facilities should have at least 8 ACH to dilute the contaminated air; as for the anteroom, it should be at least 6 ACH. Conditioned air would be supplied through a HEPA filter with a dimension of 610 mm × 310 mm. Air mixing should not be applied in this type of healthcare facility. The exhaust air is removed through a wall-mounted exhaust grille with a dimension of 1000 mm × 200 mm right behind the patient’s head. The exhaust flow rate is modulated by an inverter with variable air volume control depending on pressurization specification to establish negative pressure in the room. Exhaust air system attached with bag-in/bag-out (BI/BO) filters to ensure the exhaust air is free from infectious contaminants. The temperature of the PICU isolation room was set at 22 ± 2 °C, compiled with the AAP and ASHRAE standard. The pressurization schemes for both rooms were designed accordingly to the Taiwan CDC; a minimum value for the anteroom was set at −5 Pa, and for the isolation room, at −10 Pa.

### 2.2. CFD Simulation and Improvement Strategy

**Figure 2:**
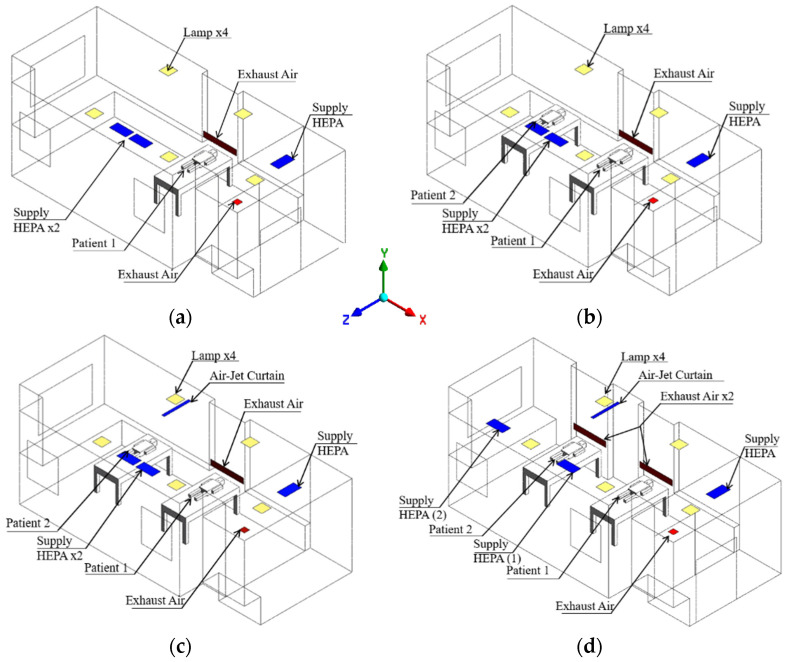
The geometry model of the isolation room based on infection control strategies: (**a**) case 1 (baseline); (**b**) case 2 (two patients); (**c**) case 3 (with air-jet curtain); (**d**) case 4 (new arrangement ventilation system).

The simulation of the PICU isolation room was performed by ANSYS Fluent Workbench Version 2020 R2 [21]. The 3D geometry model of the PICU isolation room was made based on the existing layout, as shown in Figure 2. The dimensions of length, width, and height for the isolation room were 6.8 m × 4.0 m × 2.6 m. The baseline case was supposed to be occupied by one patient. The nature scale of future pandemics cannot be adequately predicted, as it is quite a difficult challenge to predict the sufficient capacity of isolation facilities. The two-bed ward was designed to provide maximum usage of the isolation room.

A total of four cases were analyzed in this study based on the additional patient. The baseline case was supposed to be occupied by one patient; then, the case was improved with the possibility of accommodating more than one patient. Additionally, an air-jet curtain and different ventilation system arrangement layout was implemented to improve the ventilation performance and protect each patient and the medical staff from exposure to the contaminant. Various cases are described below.

- Case 1: baseline case (Figure 2a).
- Case 2: occupied by two patients (Figure 2b).
- Case 3: two patients with additional air-jet curtain ceiling mounted, placed between the patient. The corresponding velocity was 0.5 m/s (Figure 2c).
- Case 4: case 3 with a new arrangement ventilation system based on an additional exhaust air grille identical with the original design placed right besides patient 2’s head along with one HEPA placed for each patient (Figure 2d).

### 2.3. Boundary Conditions and Initial Conditions

Monitoring carbon dioxide (CO_2_) exhaled by patients can provide a valuable assessment to ensure adequate ventilation systems in adult and pediatric patients. Contaminants such as CO_2_ and other waste anesthetic gases can cause severe complications for the patients if they are not appropriately handled; elevated CO_2_ concentrations can be related to an increase in different indoor contaminant concentrations [22]. Thus, the concentration of CO_2_ generated inside the PICU isolation room can be considered as a good indicator for ventilation efficiency in the process of air renewal [23]. The CO_2_ concentration in the outdoor atmosphere was about 400 ppm [24], which was the concentration value for the supply of HEPA CO_2_. Meanwhile, permissible CO_2_ concentration inside the rooms was lower than 1000 ppm [25], so that the value of concentration at 1000 ppm was used as an initial condition in the isolation room.

Transient numerical simulations were implemented to monitor and investigate the concentration level of CO_2_ for 900 s. Table 1 shows the boundary condition of the numerical simulation. Supply air inside the isolation room was set at 0.49 m/s and anteroom at 0.47 m/s. The value of the pressure outlet was set at −11.4 Pa for the PICU isolation room. These boundaries data were taken from the field measurement that has been conducted comprehensively. During expiration, the corresponding velocity for the child during expiration is 1.12 m/s based on a numerical simulation study conducted by Tsega [26]. The CO_2_ level in the exhaled air by a human is about 38,000 ppm.

The PICU isolation room contains several parts that generate heat: the window, exterior wall, lamps, and humans. Lightings were assumed to generate a sensible heat load of 33.3 W/m^2^. The sensible heat load due to a pediatric patient was assumed 34.87 W/m^2^; heat gain of a child is taken as 75% of adult based on ASHRAE GRP 158 Cooling and Heating Load Calculation [27]. The room had a window, and the exterior wall facing the south and west were assumed to have convection heat transfer from ambient temperature with the heat transfer coefficient of 3.18 W/m^2^K and 5.6 W/m^2^K, respectively. The realizable k-ε was selected as the turbulence model, while the finite control volume method with coupled was used as the pressure–velocity coupling algorithm [28]. The grid independence test proved the numerical robustness of CFD Fluent in solving this problem, and a grid containing 1,154,759 cells was employed for the simulation.

### 2.4. Field Measurement Test and Validation

The field measurement was carried out in a new PICU negative-pressurized isolation room. The measurement data were used as the basis of the parameters as the boundary condition in CFD simulation [29]. The apparatus for field measurements was calibrated within 1-year due date for accurate and precise measurements data, as shown in Table 2. The parameters that needed to be measured and analyzed were airflow rate, pressurization, and temperature. The measurements data (shown in Table 3) were used for the boundary condition of the numerical simulation. Airflow rate was measured at the outlet of supply HEPA, which had a total value of 648 cubic meters per hour (CMH). Air changes rate per hour (ACH) was calculated from the estimated HEPA airflow rate divided by the room volume; thus, the ACH of the anteroom and PICU isolation room were 6.42 ACH and 9.32 ACH, respectively. Both values complied with the standard minimum ACH for the anteroom and isolation room. Additionally, the pressure test was conducted to meet the specific design condition of the isolation room. The anteroom was maintained at a pressure of −6.12 Pa to the corridor. Likewise, the PICU isolation room was maintained at a pressure of −11.4 to the anteroom.

**Figure 3:**
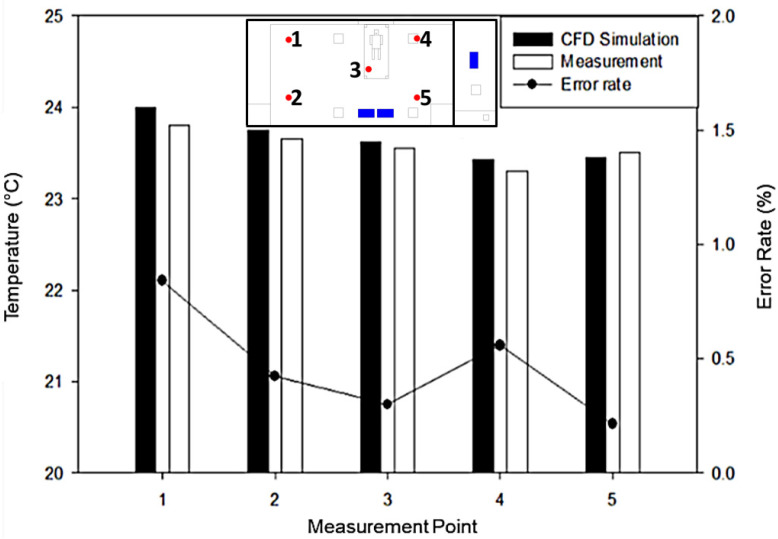
Validation between the field measurement and numerical simulation.

Field measurement was conducted comprehensively to validate the simulation results. Five points locations were monitored and measured for the temperature value. The method used for validation for the numerical simulation was the steady-state condition. As shown in Figure 3, the temperature results in numerical simulation and measurement were between 23.4 °C and 24.0 °C and complied with the standard of temperature for isolation room (22 ± 2 °C). The temperature error rate percentage result between numerical simulation and field measurement test for every measurement point was less than 1%, indicating a good agreement between the numerical and experimental data.

## 3. Results and Discussion

### 3.1. Airflow Distribution

**Figure 4:**
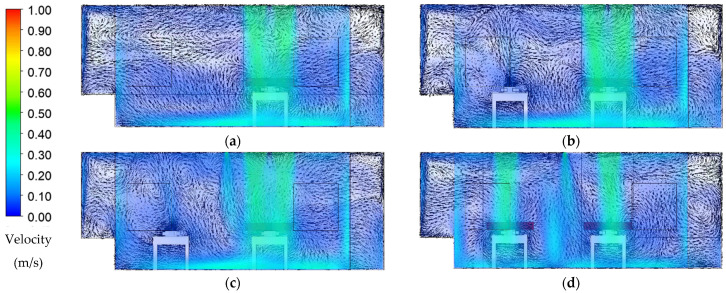
Airflow distribution pattern at 900 s: (**a**) case 1 (baseline); (**b**) case 2 (two patients); (**c**) case 3 (with air-jet curtain); (**d**) case 4 (new arrangement ventilation system).

Transient numerical simulation was conducted within 900 s by assuming the initial CO_2_ concentration level at 1000 ppm. The average CO_2_ concentration level, temperature, pressure, and airflow distribution inside the isolation room were quantified to evaluate the ventilation system performance and contaminant removal. The airflow results from the transient simulation at 900 s for each case are presented in the form of velocity contour superimposed with velocity vectors (Figure 4). Plane XY at −3.7 m illustrated the cross-section at the patient mouth area. Case 1 shows (Figure 4a) that two HEPA located across the patient induce laminar airflow and then extend downward after leaving the supply air before it spreads out thoroughly in the PICU isolation room. The arrangement of the exhaust air grille located right behind the patient’s head was realized to be beneficial for infection control. Velocity vector exhaled by the patient can be seen leaving directly through the exhaust air without entrainment into airstream supply. Some vortex vector fields can be found on the corner right of the room, which highlights the possibility of accumulating contaminants in that region.

Figure 4b shows a vector distribution pattern for the configuration of the PICU isolation room occupied by two patients. Overall, air distribution from supply HEPA shows the same pattern as the baseline case. There is an additional exhaled airstream formed by patient 2 on the left side of the room. The vortex vector field could be found in some regions above the patient’s head since there is no exhaust air nearby. Hence, the airstream from patient 2 mixes with the air supply of HEPA, thus spreading inside the room before it can exit through the exhaust air grille near-patient 1. The air-jet curtain is added between the patients to keep each patient safe from the contaminated exhaled air, as shown in Figure 4c. This configuration separates each patient exhaled zone and does not contaminate the HEPA airstream supply. However, the vortex vector field still appeared since there was not any exhaust air nearby. Thus, a new arrangement ventilation system layout was proposed accordingly for case 4, as shown in Figure 4d. Two HEPAs placed across patient 1 in baseline case were changed into one HEPA for each patient. Additional exhaust air grille identical with the original design was added beside patient 2 head. The result revealed that the velocity vector distribution was similar to the baseline case in this configuration of two patients. One HEPA and one exhaust air grille placed behind the patient head with an additional air-jet curtain show the possible results of handling and accommodating two patients.

### 3.2. Temperature Distribution

**Figure 5:**
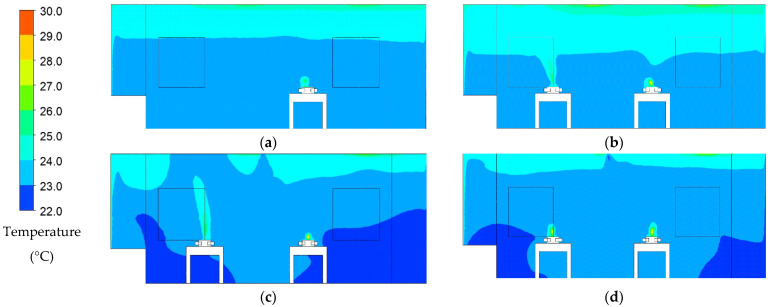
Temperature distribution pattern at 900 s: (**a**) case 1 (baseline); (**b**) case 2 (two patients); (**c**) case 3 (with air-jet curtain); (**d**) case 4 (new arrangement ventilation system).

The temperature distribution results from the transient simulation at 900 s for each case are presented in color contour, as illustrated in Figure 5. The contour on the ceiling was slightly higher than in another region due to heat generation from the lightings. The left side of the room contained the wall and window that was exposed to the sun. The higher temperature contour region above the patient’s head was the exhaled air by them. The temperature of exhaled air was set around 35 °C. The average temperature on each case was observed on this plane. During 900 s simulation, the temperature average result on each case was at 23.32 °C, 23.42 °C, 23.23 °C, and 23.72 °C. Without the additional patients, it was revealed that the baseline case had a lower temperature than case 2. The air-jet curtain improved the temperature distribution in case 3. In case 4, the results show it has the highest temperature average comparing to others. This may be due to an additional exhaust air grille for patient 2. As a result, it removes more cold air from supply HEPA. Overall, all the cases reached the standard temperature for the isolation room, which was around 22 ± 2 °C.

### 3.3. Concentration Distribution and Concentration Decay

**Figure 6:**
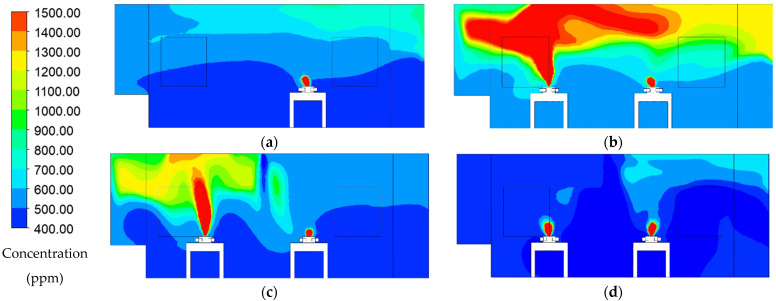
Concentration distribution pattern at 900 s: (**a**) case 1 (baseline); (**b**) case 2 (two patients); (**c**) case 3 (with air-jet curtain); (**d**) case 4 (new arrangement ventilation system).

Figure 6 illustrates CO_2_ concentration distribution at 900 s for each case. Case 1 shows (Figure 6a) a higher concentration of CO_2_ accumulation above the patient’s head indicates the exhaled air pathway. The vortex vector field that generates on the top right of the room makes the concentration higher in this region. Case 2 (Figure 6b) illustrates that the area that formed higher concentrations showed more intense CO_2_ accumulation than case 1. The higher concentration accumulated above patient 2 head, and it could spread out along with exhaled air pathway from that patient. Hence, the CO_2_ concentration on the top right region of the room would further get affected. Case 3 (Figure 6c) illustrates that CO_2_ concentration from patient 2 only accumulates above patient 2 head without spreading too much to another patient. The air-jet curtain would separate the mixing of air exhaled from patient 2 to the air supply stream and create protection for each patient. However, this additional configuration did not significantly improve the ventilation performance and dilution process. CO_2_ concentration distribution shows the optimized result in case 4, as shown in Figure 6d. A higher concentration of CO_2_ only forms around the patient mouth region, and then, it directly exits through exhaust air placed behind each patient’s head. It indicates there is no recirculation of exhaled air from the patient into the supply airstream. This arrangement can help to reduce the CO_2_ concentration within the room.

**Figure 7:**
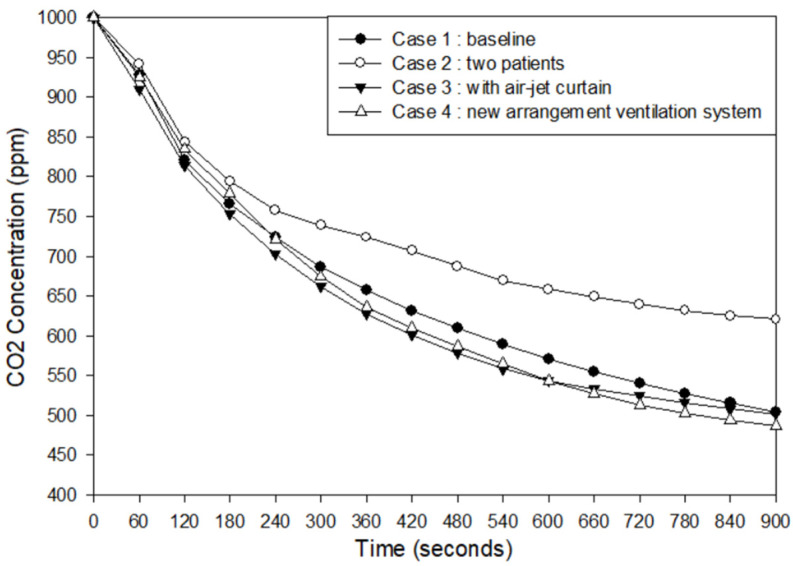
Concentration decay results.

Concentration decay is observed in Figure 7. The average concentration decay was observed on plane XZ at 1.2 m, the possible height of exhaled air from the patient, and likely the accumulated contaminant. Based on the numerical simulation result, the concentration can be diluted to 504.10 pm on case 1 occupied by one patient. If two patients occupied the room, the result would have a higher value at 620.4 ppm. The air-jet curtain was added in case 3 to improve the dilution process of case 2, which can reduce the concentration to 501.60 ppm, even better than the baseline case. As for case 4, the results show it has a better performance than the others on removing the contaminant, which can dilute concentration to 486.50 ppm. It indicates that the isolation room is feasible for two patients with a new ventilation system arrangement by placing one HEPA across the patient, exhaust air grille behind the patient head, and optimized with an additional air-jet curtain.

### 3.4. Particle Tranjectories

Based on another study, a coughing activity could generate droplets that travel between 6 and 28 m/s and have the diameter range between 50 μm and 400 μm. The further Aerosol model has been implemented to simulate the motion of particles exhaled by a coughing patient to see the particle trajectories from the patient. The effect of the particle spread coming from a coughing patient is modeled using the discrete phase model (DPM). Transient numerical simulation conducted for 100 s duration, and the patient’s coughing lasts for 2 s starting at 2 s. A velocity of 10 m/s with minimal diameter distribution of 74 μm and maximal diameter distribution of 500 μm was used to define the initial condition of cough droplets in this simulation. 

**Figure 8:**
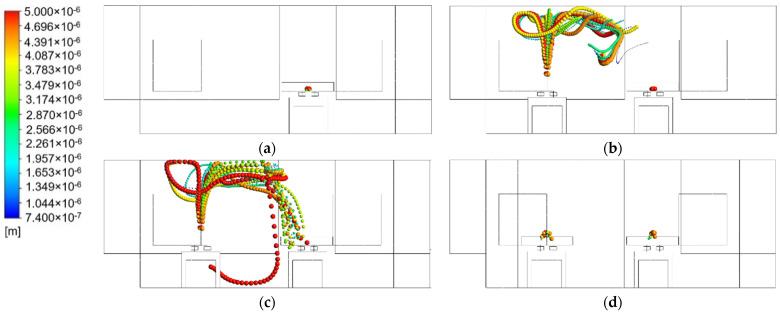
Particle trajectories at 0–100 s: (**a**) case 1 (baseline); (**b**) case 2 (two patients); (**c**) case 3 (with air-jet curtain); (**d**) case 4 (new arrangement ventilation system).

The result of the particle trajectory pathway from the patient for the transient simulation is shown in Figure 8. The particle emitted by the coughing patient for the baseline case is shown in Figure 8a during the 2–100 s simulation. Aerosol particles from the cough droplet leave through the exhaust air grille directly without being dispersed inside the room due to the location of exhaust air behind the patient’s head. Case 2 occupied by two patients is presented in Figure 8b. Patient 2 would also emit cough droplets with the identical setup as the baseline patient. It can be observed from 2 s to 100 s that both of them could be exposed to the aerosol particle emitted from patient 2. The particle trajectory streams would be carried by supply airstream and dispersed inside the room before eventually leaving through the exhaust air grille. It was observed that the medium to small size particles still entered the supply airstream.

The particle trajectory from the coughing patient with an additional air-jet curtain is illustrated in Figure 8c. The air-jet curtain would help some of the particles emitted from patient 2 to distribute into the exhaust air grille near-patient 1, but some of the particles are still left behind and dispersed inside the room. It can be observed that this configuration is still not optimized to decrease the aerosol particle since there is still a possibility of exposure to the other patient and medical staff inside. Exposure to infectious aerosols that have been emitted to the isolation room by the patient can cause a harmful transmission during the movement of supply air to the exhaust. Figure 8d shows the best result on removing particles from coughing by two patients. The new arrangement of ventilation system could contain two patients and remove the emitted particle from both of them. Like the baseline case, there is no particle left behind since it directly leaves through the exhaust air grille behind the patient’s head.

## 4. Conclusions

In this study, numerical transient simulation of pediatric intensive care units (PICU) isolation rooms was carried out to evaluate a better ventilation performance dealing with two patients. The baseline case had two HEPA located across the patient and exhaust air located behind the patient’s head, which was beneficial for infection control purposes. As for case 2, the air exhaled by patient 2 would entrain back into the supply airstream, resulting in higher CO_2_ concentrations around both patients. We proposed an air-jet curtain with a velocity of 0.5 m/s to improve the ventilation system. The results show that this configuration can reduce concentration decay. However, no exhaust air grille near-patient 2 created a higher concentration above the patient’s head. Thus, a new arrangement ventilation system layout was proposed accordingly. Two HEPAs placed across patient 1 in baseline case were changed into one HEPA for each patient. Additional exhaust air grille identical to the original design was added behind patient 2′s head. During the 900 s duration, the CO_2_ was reduced to 504 ppm for case 1; 620 ppm for case 2; 501 ppm for case 3; and 486 ppm for case 4. The results reveal that case 4 could accommodate two patients with a feasible ventilation system to protect the medical staff and both patients inside.

## Figures and Tables

**Table 1:** Boundary conditions for numerical simulation.

Parameter	Type	Value
Supply air	Velocity inlet	Isolation room HEPA: 0.49 m/s Anteroom HEPA: 0.47 m/s Temperature: 22.2 °C Concentration: 400 ppm
Exhaust air	Pressure outlet	Temperature: 24 °C Isolation room pressure: −11.4 Pa Anteroom pressure: −6.12 Pa
CO_2_ concentration	Velocity inlet	Velocity inlet: 1.12 m/s Temperature: 35 °C Patient’s exhale: 38,000 ppm
Patient	Wall	Heatflux: 34.87 W/m^2^
Lightings	Wall	Heatflux: 33.3 W/m^2^
Windows	Wall	Heat transfer coefficient: 3.18 W/m^2^K
Walls	Wall	Heat transfer coefficient: 5.60 W/m^2^K

**Table 2:** Apparatus for the field measurement test.

Apparatus Model	Parameters	Operative Range	Accuracy
TSI-8380	Velocity Pressure	0.125–12.5 (m/s) Diff ± 3735 Pa	± 3% ± 2%
TSI TA465P	Temperature Humidity	−10~60 °C 5~95% RH	± 0.3 °C ± 1%

**Table 3:** Measurement data.

Room Name	Volume (m^3^)	Inlet/Outlet	Airflow Rate (CMH)	Pressure (Pa)	Air Changes Hour (ACH)
Anteroom	19.15	HEPA 1 EA 1	123 315	−6.12	6.42
Isolation room	69.5	HEPA 2 HEPA 3 EA 2	331 317 1318	−11.4	9.32

## Data Availability

The data presented in this study are available on request from the corresponding author.
